# Association between TCF7L2 Gene Polymorphism and Cancer Risk: A Meta-Analysis

**DOI:** 10.1371/journal.pone.0071730

**Published:** 2013-08-09

**Authors:** Jingxiang Chen, Tao Yuan, Menggang Liu, Ping Chen

**Affiliations:** 1 Department of Hepatobiliary Surgery, The Third Affiliated Hospital of the Third Military Medical University, Chongqing, China; 2 Department of Hepatobiliary Surgery, Jiangjin Central Hospital, Chongqing, China; Virginia Commonwealth University, United States of America

## Abstract

**Objective:**

The transcription factor 7-like 2 (*TCF7L2*) gene has been suggested to play an important role in the pathogenesis of cancer. However, the results have been inconsistent. In this study, we performed a meta-analysis to clarify the associations between *TCF7L2* polymorphism and cancer risk.

**Methods:**

Published literature from PubMed and EMBASE were retrieved. Pooled odds ratios (ORs) with 95% confidence interval (CIs) were calculated using fixed- or random-effects model.

**Results:**

A total of 19 studies (14,814 cases and 33,856 controls) were identified for the analysis of the association between *TCF7L2* polymorphism and cancer risk. The results showed that *TCF7L2* polymorphism was associated with breast cancer (Homogeneous model: OR = 1.17, 95%CI = 1.02–1.35, *I* ^2^ = 21.8%, *p* for heterogeneity = 0.276; Heterogeneous model: OR = 1.11, 95%CI = 1.03–1.20, *I* ^2^ = 0.0%, *p* for heterogeneity = 0.543), prostate cancer (Homogeneous model: OR = 0.89, 95%CI = 0.84–0.96, *I* ^2^ = 0.0%, *p* for heterogeneity = 0.640; Heterogeneous model: OR = 0.89, 95%CI = 0.84–0.95, *I* ^2^ = 0.0%, *p* for heterogeneity = 0.871), and colon cancer (Heterogeneous model: OR = 1.15, 95%CI = 1.01–1.31, *I* ^2^ = 0.0%, *p* for heterogeneity = 0.658), but not with colorectal cancer, lung cancer, and ovarian cancer.

**Conclusions:**

The present meta-analysis indicated that there were significantly associations between the *TCF7L2* rs7903146 polymorphism and risk of breast, prostate and colon cancers, rather than colorectal cancer, lung cancer, and ovarian cancer.

## Introduction

The transcription factor 7-like 2 (*TCF7L2*) gene, previously reported as *TCF-4*, has been found to be associated with type 2 diabetes. Rs7903146 variant of *TCF7L2* gene was firstly identified as one susceptibility marker of type 2 diabetes by genome-wide association study [1]. The following studies further confirmed the association between *TCF7L2* rs7903146 variant and type 2 diabetes [2]. In addition, individuals carrying T alleles of *TCF7L2* rs7903146 variant demonstrated high risk of insulin resistance [3]. Alternatively, *TCF7L2* may affect cancer independently of diabetes, as the *TCF7L2* gene product is involved the Wnt/β-catenin signaling pathway. TCF7L2 forms an active nuclear complex with β-catenin that binds and induces the expression of target genes involved in cellular proliferation, evasion of apoptosis, and tissue invasion and metastasis.

To date, many studies have been published investigating the association between *TCF7L2* rs7903146 or rs12255372 (it is in high linkage disequilibrium with rs7903146) and several types of cancer, including breast cancer [4]–[8], prostate cancer [5], [9]–[12], colorectal cancer [5], [13]–[15], colon cancer [5], [16], lung cancer [5] and ovarian cancer [6]. However, the conclusions have been conflicting. Therefore, we performed a meta-analysis to clarify the association between *TCF7L2* rs7903146 variant and cancer risk.

## Materials and Methods

### Literature and Search Strategy

We searched the PubMed and EMBASE literature databases. The search strategy to identify all possible studies involved the use of the following key words: (*TCF7L2* or transcription factor 7-like 2 or *TCF-4*) and (variant or variation or polymorphism or genotype) and (cancer or carcinoma or tumor). All related studies published in English language were included. The reference lists of retrieved articles were hand-searched. If more than one article were published using the same case series, only the study with the latest data was included. The literature search was updated on February 18, 2013.

### Inclusion Criteria and Data Extraction

The studies included in the meta-analysis must meet all the following inclusion criteria: (1) evaluates the associations of *TCF7L2* polymorphism with cancer risk; (2) uses case–control or cohort design; and (3) provides sufficient data for calculation of odds ratio (OR) with 95% confidence interval (CI). The following information was extracted from each study: (1) name of the first author; (2) year of publication; (3) country of origin; (4) ethnicity; (5) cancer type; (6) sample size of cases and controls; (7) covariates’ adjusted OR with 95%CI under co-dominant model; (8) minor allele frequency in cases and controls; (9) *p* for Hardy Weinberg Equilibrium test in controls; and (10) studied polymorphism. The two authors independently assessed the articles for compliance with the inclusion/exclusion criteria, resolved disagreements and reached a consistent decision.

### Statistical Analysis

The association of *TCF7L2* polymorphism with cancer risk was estimated by calculating pooled ORs and 95%CIs under a co-dominant model. The significance of pooled OR was determined by Z test (P<0.05 was considered statistically significant). Q test was performed to evaluate the between-study heterogeneity. A random- (DerSimonian-Laird method [17] or fixed- (Mantel–Haenszel method) [18] effects model was used to calculate pooled OR in the presence (*P*≤0.10) or absence (*P*>0.10) of heterogeneity, respectively. Subgroup analysis by cancer type was performed to address the between-study heterogeneity. Publication bias was assessed by Begg’s test [19] and Egger’s test [20] (*P*<0.05 was considered statistically significant). Data analysis was performed using STATA version 11 (StataCorp LP, College Station, TX, USA).

## Results

### Characteristics of the Studies

**Figure 1:**
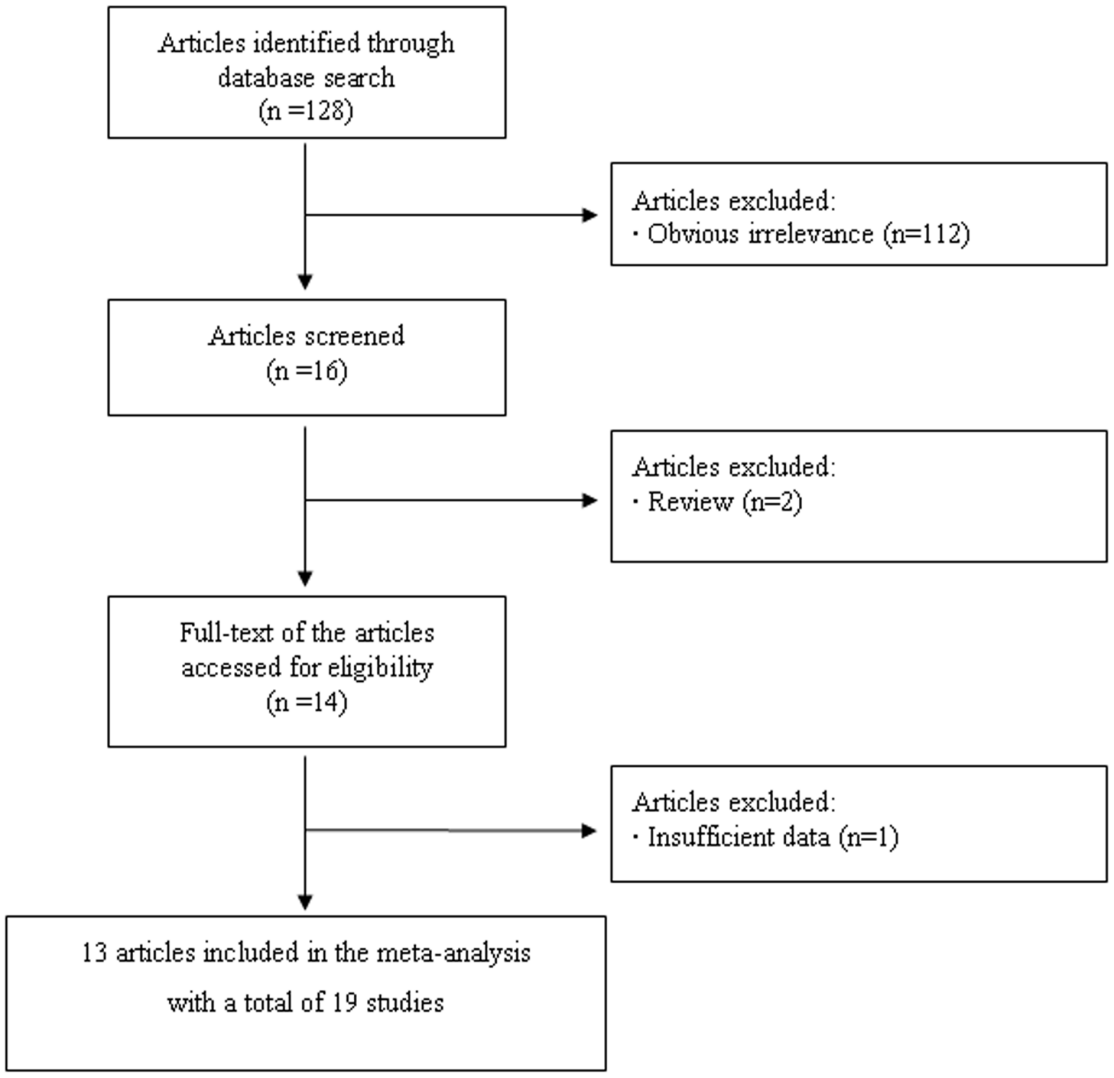
Flow chart of meta-analysis for exclusion/inclusion of individual articles (or studies).

In this study, we followed the PRISMA Statement (Checklist S1). A flow chart describing the process of inclusion/exclusion of study is presented in Fig. 1. The literature search identified a total of 128 potentially relevant papers. Of them, 112 papers were excluded because of obvious irrelevance by reading the titles and abstracts. In addition, two papers were excluded because they were reviews. Then, 14 papers met the primary inclusion criteria. However, one paper was excluded because it did not provide sufficient data to calculate OR with 95%CI [21]. Since more than one study was included in two articles by Folsom et al [5] and Goode et al [6], they were considered as separate studies in the meta-analysis. In addition, since rs7903146 was in high LD with rs12255372, we combined all the included studies together. At last, 19 studies for the association between *TCF7L2* polymorphism and cancer risk were included in the final meta-analysis. Of them, five were on breast cancer, five were on prostate cancer, four were on colorectal cancer, two were on colon cancer, two were on lung cancer, and one was on ovarian cancer. The characteristics of the included studies are listed in Table 1. It should be noted that because most studies provided covariates’ adjusted OR with 95%CI under a co-dominant model, we calculated the pooled estimate under this model only.

**Table 1:** Characteristics of included studied of association between *TCF7L2* rs7903146 (or its proxy rs12255372) polymorphism and cancer risk.

Study	Country	Ethnicity	Cancer type	Sample size		Homogeneous co-dominant model			Heterogeneous co-dominant model			MAF		*P* for HWE	Studied SNP
				Cases	Controls	OR^a^	95% CI		OR^a^	95% CI		Cases	Controls		
Burwinkel,2006	Germany	European	Breast cancer	592	735	1.37	0.91	2.08	1.21	0.97	1.53	0.29	0.26	0.643	rs12255372
Agalliu,2008	USA	European	Prostate cancer	582	540	0.73	0.44	1.20	0.94	0.72	1.23	0.27	0.29	0.777	rs12255372
Folsom,2008a	USA	Mixed	Colorectal cancer	180	11410	1.56	0.97	2.53	1.17	0.85	1.61	0.34	0.29	0.806	rs7903146
Folsom,2008b	USA	Mixed	Colon cancer	128		2.15	1.27	3.64	1.25	0.85	1.83	0.36	0.29	0.806	rs7903146
Folsom,2008c	USA	European	Lung cancer	177		1.59	0.96	2.63	1.63	1.17	2.25	0.37	0.29	0.806	rs7903146
Folsom,2008d	USA	Black	Lung cancer	62		0.62	0.22	1.76	0.75	0.44	1.3	0.26	0.29	0.806	rs7903146
Folsom,2008e	USA	Mixed	Breast cancer	342		0.87	0.57	1.32	0.98	0.78	1.23	0.28	0.29	0.806	rs7903146
Folsom,2008f	USA	Mixed	Prostate cancer	366		1.04	0.72	1.50	0.80	0.64	0.99	0.27	0.29	0.806	rs7903146
Hazra,2008	USA	European	Colorectal cancer	357	814	0.63	0.37	1.08	0.90	0.68	1.18	0.25	0.29	0.497	rs12255372
Slattery,2008	USA	Mixed	Colon cancer	1573	1962	1.03	0.80	1.33	1.14	0.99	1.31	0.29	0.28	0.878	rs7903146
Goode,2009a	USA	European	Breast cancer	779	830	0.92	0.62	1.36	1.16	0.93	1.43	NA	0.28	0.23	rs12255372
Goode,2009b	USA	European	Ovarian cancer	391	458	0.97	0.56	1.68	0.95	0.71	1.26	NA	0.26	0.96	rs12255372
Tsilidis,2009	USA	Mixed	Colorectal cancer	202	354	1.44	0.81	2.57	1.13	0.78	1.66	0.32	0.28	0.196	rs7903146
Wang,2009	USA	Mixed	Prostate cancer	249	249	0.62	0.32	1.20	0.94	0.62	1.42	0.27	0.30	0.151	rs7903146
Meyer,2010	USA	Mixed	Prostate cancer	365	5757	0.88 ^b^	0.75	1.03	0.88 ^b^	0.75	1.03	NA	0.30	>0.05	rs7903146
Machiela,2012	USA	European	Prostate cancer	2782	4458	0.90 ^b^	0.83	0.97	0.90 ^b^	0.83	0.97	NA	0.28	>0.05	rs7903146
Naidu,2012	Malaysia	Southeast Asian	Breast cancer	387	252	1.574	0.829	2.987	1.329	0.948	1.862	0.31	0.26	0.518	rs12255372
Sainz,2012	Germany	European	Colorectal cancer	1764	1749	1.28	1.01	1.63	1.13	0.98	1.30	0.31	0.28	0.435	rs7903146
Connor,2012	USA	Mixed	Breast cancer	3523	4209	1.24	1.03	1.49	1.09	0.99	1.20	0.26	0.23	0.121	rs7903146

a Covariates’ adjusted estimate ^b^ Estimate under an additive model.

MAF, minor allele frequency; NA, not available; HWE, Hardy Weinberg Equilibrium.

### Meta-analysis Results

**Figure 2:**
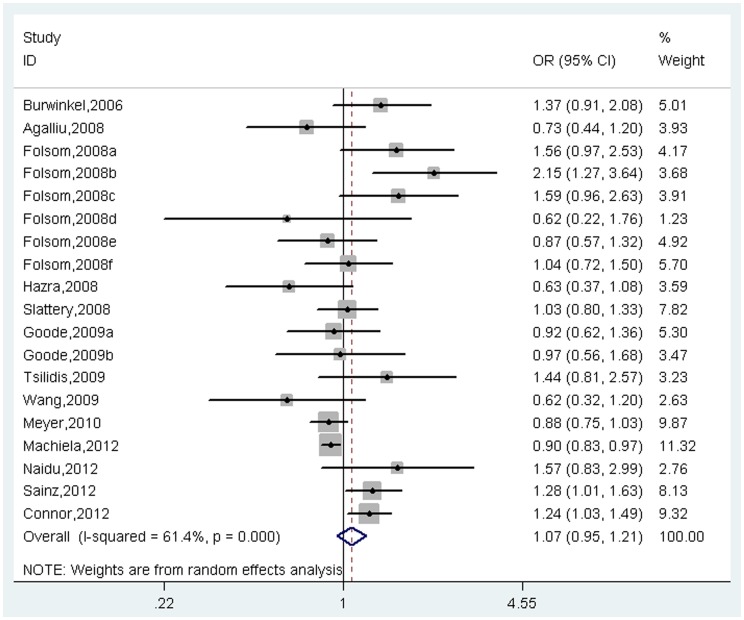
Meta-analysis of association between *TCF7L2* rs7903146 polymorphism and cancer risk under homogeneous co-dominant model.

**Figure 3:**
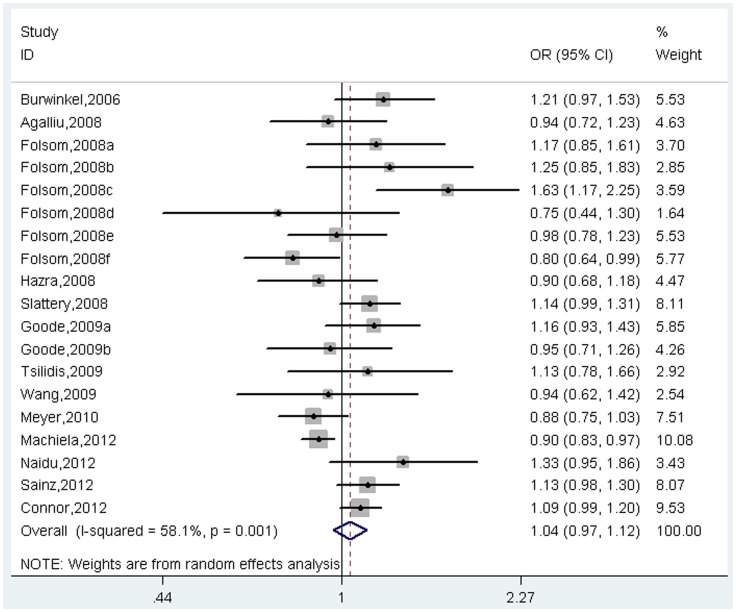
Meta-analysis of association between *TCF7L2* rs7903146 polymorphism and cancer risk under heterogeneous co-dominant model.

A total of 14814 cases and 33856 controls were identified for the analysis of the association between *TCF7L2* polymorphism with cancer risk. The results indicated that *TCF7L2* polymorphism might not be associated with cancer risk under co-dominant model (Homogeneous model: OR = 1.07, 95%CI = 0.95–1.21, *I* ^2^ = 61.4%, *p* for heterogeneity<0.001, Figure 2; Heterogeneous model: OR = 1.04, 95%CI = 0.97–1.12, *I* ^2^ = 58.1%, *p* for heterogeneity = 0.001, Figure 3). However, further subgroup analysis by cancer type suggested that the effect size was significant for breast cancer (Homogeneous model: OR = 1.17, 95%CI = 1.02–1.35, *I* ^2^ = 21.8%, *p* for heterogeneity = 0.276; Heterogeneous model: OR = 1.11, 95%CI = 1.03–1.20, *I* ^2^ = 0.0%, *p* for heterogeneity = 0.543), prostate cancer (Homogeneous model: OR = 0.89, 95%CI = 0.84–0.96, *I* ^2^ = 0.0%, *p* for heterogeneity = 0.640; Heterogeneous model: OR = 0.89, 95%CI = 0.84–0.95, *I* ^2^ = 0.0%, *p* for heterogeneity = 0.871), and colon cancer (Heterogeneous model: OR = 1.15, 95%CI = 1.01–1.31, *I* ^2^ = 0.0%, *p* for heterogeneity = 0.658), but not for colorectal cancer, lung cancer, and ovarian cancer (all *p*>0.05, Table 2).

**Table 2:** Pooled ORs and 95%CIs of the association between *TCF7L2* rs7903146 (or its proxy rs12255372) polymorphism and cancer risk.

Contrasts	No. of studies (cases/controls)	Homogeneous co-dominant model				Heterogeneous co-dominant model			
		OR	95%CI	*I* ^2^ (%)	*P*_H_	OR	95%CI	*I* ^2^ (%)	*P*_H_
All	19 (14814/33856)	1.07	0.95–1.21	61.4	<0.001	1.04	0.97–1.12	58.1	0.001
Cancer type									
Breast cancer	5 (5623/17436)^a^	1.17	1.02–1.35	21.8	0.276	1.11	1.03–1.20	0.0	0.543
Prostate cancer	5 (4358/22493)^a^	0.89	0.84–0.96	0.0	0.640	0.89	0.84–0.95	0.0	0.871
Colorectal cancer	4 (2502/14327)^a^	1.18	0.84–1.66	59.1	0.062	1.09	0.98–1.22	0.0	0.507
Colon cancer	2 (1710/13372)^a^	1.43	0.70–2.94	83.6	0.014	1.15	1.01–1.31	0.0	0.658
Lung cancer	2 (239/11410)^a^	1.11	0.45–2.73	60.8	0.110	1.14	0.53–2.44	82.7	0.016
Ovarian cancer	1 (391/458)	0.97	0.56–1.68	–	–	0.95	0.71–1.27	–	–

a Shared the same number of controls (*n* = 11410).

### Potential Publication Bias

No publication bias could be detected under homogeneous co-dominant model (*p* = 0.780 for Begg’s test and *p* = 0.123 for Egger’s test) and heterogeneous co-dominant model (*p* = 0.889 for Begg’s test and *p* = 0.274 for Egger’s test).

## Discussion

To the best of our knowledge, our meta-analysis represents the first one investigating the association between *TCF7L2* rs7903146 polymorphism and cancer risk. The results suggested that *TCF7L2* rs7903146 polymorphism might not be associated with cancer risk. However, further stratified analysis demonstrated the significant association with breast cancer, prostate cancer and colon cancer, rather than colorectal cancer, lung cancer, and ovarian cancer.

Interestingly, the T allele of rs7903146 polymorphism, which has been associated with increased risk of type 2 diabetes, showed the inverse association with prostate cancer based on our meta-analysis. The finding was consistent with those from the individual studies by Folsom et al. [5] and Machiela et al [12]. However, the other three included studies did not suggest any association [9]–[11]. In addition, we found positive association between rs7903146 polymorphism and breast cancer risk. Recently, Michailidou K et al. [22] have found rs7904519 in intron 4 of *TCF7L2* (r^2^ = 0.37 with rs7904519/rs12255372), to be associated with breast cancer. Further studies are necessary to examine the potential different mechanisms of *TCF7L2* polymorphism in various cancers.

Heterogeneity between studies is common in meta-analysis of genetic association studies. Although heterogeneity can be taken into account by performing a random-effects model, it would increase the odds of type I error [23]. We found significant between-study heterogeneity in the association of *TCF7L2* rs7903146 polymorphism with cancer risk. Therefore, subgroup analysis by cancer types was performed to explore the source of heterogeneity. The results showed that the between-study heterogeneity disappeared in several subgroups, but remained in other subgroups suggesting other covariates might confound the association.

The current meta-analysis has several strengths. First, OR (95% CI) after covariates adjustment from individual study was used to calculate the pooled the estimate, which increased the accuracy of effect estimate. Second, statistical power was greatly improved for the association study of *TCF7L2* rs7903146 polymorphism in the pooled analysis. However, several limitations should also be noted. First, the case – control study design does not allow for the inference of causality between the gene polymorphism and the outcome. Second, the effect of gene – gene/gene – environment interactions was not addressed in this meta-analysis. Third, although ethnicity plays an important role in the association of *TCF7L2* rs7903146 polymorphism with cancer risk, we did not perform the further subgroup analysis by ethnicity because of limited studies for each cancer type.

In conclusion, the results indicated that there was a significantly association between *TCF7L2* rs7903146 polymorphism and the risk of breast cancer, prostate cancer and colon cancer, rather than colorectal cancer, lung cancer, and ovarian cancer. Further well-designed large-scale studies with the consideration of gene–gene and gene–environment interactions should be conducted to investigate the association in future.

## Supporting Information

Checklist S1 **PRISMA Checklist.**
(DOC)
